# The Relationship between School Infrastructure and School Nutrition Program Participation and Policies in New York City

**DOI:** 10.3390/ijerph19159649

**Published:** 2022-08-05

**Authors:** Melissa Pflugh Prescott, Judith A. Gilbride, Sean P. Corcoran, Brian Elbel, Kathleen Woolf, Roland O. Ofori, Amy Ellen Schwartz

**Affiliations:** 1 Department of Food Science and Human Nutrition, University of Illinois at Urbana-Champaign, 539 Bevier Hall, 905 S. Goodwin Ave., Urbana, IL 61801, USA; 2 Department of Nutrition and Food Studies, Steinhardt School of Culture, Education, and Human Development, New York University, 411 Lafayette Street, 5th Floor, New York, NY 10003, USA; 3 Department of Leadership, Policy, and Organization, Vanderbilt University, 230 Appleton Place, PMB 414, Nashville, TN 32703, USA; 4 Department of Population Health, Grossman School of Medicine, New York University, 227 East 30th Street, New York, NY 10016, USA; 5 Maxwell School of Citizenship and Public Affairs, Syracuse University, 426 Eggers Hall, Syracuse, NY 13244, USA

**Keywords:** school nutrition programs, meal participation, untraditional lunch periods, open campus, co-location, overcrowding, cafeteria infrastructure, kitchen infrastructure

## Abstract

School nutrition programs (SNP) provide much needed access to fruits, vegetables, and other healthy foods at low or no cost. Yet, the infrastructure of school kitchens and cafeteria vary across schools, potentially contributing to systematic barriers for SNP operation and equity. The purpose of this paper is to examine the association between school infrastructure and outcomes including meal participation, untraditional lunch periods, and having an open campus. Regression analyses were conducted using administrative data for 1804 schools and school nutrition manager survey data (*n* = 821) in New York City (NYC). Co-location was significantly associated with open campus status (OR = 2.84, CI: 1.11, 7.26) and high school breakfast participation (β = −0.056, *p* = 0.003). Overcrowding was associated with breakfast (elementary: β = −0.046, *p* = 0.03; middle: β = 0.051, *p* = 0.04; high: β = 0.042, *p* = 0.04) and lunch participation (elementary: β = −0.031, *p* = 0.01) and untraditional lunchtimes (elementary: OR = 2.47, CI: 1.05, 5.83). Higher enrollment to cafeteria capacity ratios was associated with breakfast (elementary: β = −0.025, *p* = 0.02) and lunch (elementary: β = −0.015, *p* = 0.001; high: β = 0.014, *p* = 0.02) participation and untraditional lunchtimes (middle: OR = 1.66, CI: 1.03, 2.68). Infrastructure characteristics are an important source of variation across NYC schools that may hinder the equity of school nutrition programs across the city.

## 1. Introduction

School nutrition programs ensure that U.S. public school students have access to healthy, affordable food. Effective in 2012, the Healthy, Hunger-Free Kids Act (HHFKA) of 2010 resulted in widespread scrutiny of the integrity of school nutrition standards. Changes to school nutrition programs included weekly requirements for specific vegetable subgroups to ensure variety and calorie limits to promote healthy portion sizes [1]. Less attention was given to other factors that may also influence the success of school nutrition programs, like the space and infrastructure of school kitchens and cafeterias. Some public health advocates assert that many schools do not have the capacity to implement the new nutrition guidelines mandated by the 2010 legislation [2]. For example, many school kitchens were built to heat and hold foods, not to cook meals from scratch or to use complicated meal preparation techniques. School food preparation capacities vary from full-service kitchens equipped to cook and serve meals on site, to satellite kitchens limited to receiving plated meals from off-site full-service kitchens. Differences in kitchen capabilities may influence the appearance or perceived quality of school food. Furthermore, satellite kitchens are associated with lower school meal participation [3].

**Figure 1:**
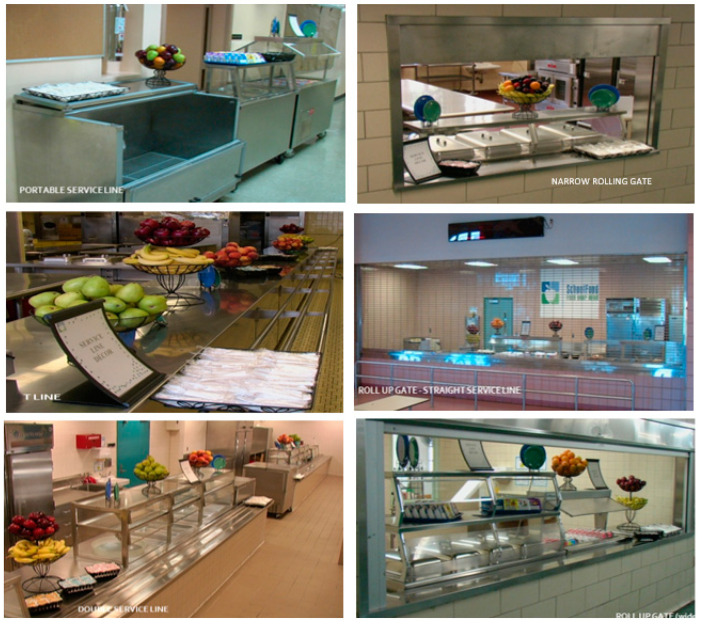
Service Line Types in New York City. From top left to bottom right: Portable Service Line, Narrow Rolling Gate, T-Line, Straight Roll-up Gate, Double Service Line and Wide Roll-up Gate were the six serving line types available to NYC public schools in 2013–2014. Photo source: NYC Office of Food and Nutrition Services.

Another key factor associated with school meal participation is the time available for students to eat [3,4,5,6]. In New York State, Chapter 296 of the Laws of 1994 amended Education Law requires all public schools to schedule a “reasonable time” for all full-day students in grades pre-kindergarten to twelve to eat lunch. However, the definition of reasonable time is not stipulated and is left to the discretion of school districts and/or school principals [7]. Cafeteria service line and seating area characteristics appear to influence the time students have available to eat. Increasing the number of service lines is one method to reduce the amount of time students spend in line [8,9]. In addition, service line type may influence the amount of time students spend in line during each meal period. Service line types include portable service lines, narrow rolling gates, t-lines, straight roll-up gates, double service lines, and wide roll-up gates (Figure 1). The influence of service line types on the time available to eat and/or meal participation has not been previously studied.

Cafeteria capacities quantify the number of students that a school can safely accommodate in the seating area at one time as determined by district-level departments of education. Inadequate cafeteria capacities have been implicated as a major reason for student nonparticipation in school lunch [6], but may also shape other aspects of school meals. Schools with high student enrollments and limited cafeteria seating capacities may need to resort to increasing the number of daily lunch periods to adequately accommodate students, forcing some students to have lunch as early as 10:30 a.m. or as late as 2:00 p.m. Cafeteria space may be particularly constrained at schools with a high percentage of low-income students, who are more likely to participate in school nutrition programs [10,11]. Additionally, school principals may choose to allow their students to have an open campus policy in an effort to reduce the number of students using the cafeteria during each lunch period. Open campuses permit students to eat lunch at home or at local restaurants and corner stores instead of in the school cafeteria [12]. Open campus policies are associated with decreased school lunch participation [3,6]. Seating availability and/or the number of service lines may influence students to eat lunch off campus rather than participate in school meals. However, these relationships have not been previously studied.

Overcrowding and/or co-location of schools may also impact meal time constraints and subsequently influence school meal participation, open campus status, and/or untraditional meal times. School co-location occurs when two or more schools are housed within the same school building. Co-located schools typically share common spaces, such as cafeterias. An estimated 66% of NYC public schools were co-located during the 2020–2021 school year, and co-location has become increasingly commonplace in other school systems [13]. A qualitative assessment of school co-location concluded that co-location results in complex scheduling of meal periods to allow shared use of the cafeteria, and may have unintended consequences such as abbreviated lunch periods and untraditional lunchtimes [14].

While a handful of studies suggest a potential relationship between the infrastructure characteristics of schools and meal participation [3,6,8,9,15,16], more research is needed to understand the relationship between school meal participation and the number of service lines, type of service line, cafeteria capacity and kitchen type post-implementation of HHFKA. Systematic variations in the characteristics of school kitchens and cafeterias may help to explain differences in meal program participation across schools. While it is plausible that school infrastructure may influence the likelihood of open campus policies and untraditional meal times, little evidence is available to support this claim.

The purpose of this study is to answer the following research questions: (1) What is the association between school infrastructure characteristics and school meal participation in NYC public schools? (2) Do school infrastructure characteristics predict the likelihood of having untraditional lunch periods (lunch before 11:00 a.m. or after 1:30 p.m.) in NYC public schools? (3) Do school infrastructure characteristics predict the likelihood of an open campus lunch policy in NYC public schools? For this study, infrastructure characteristics include school co-location status, building overcrowding status, type of service line in use, number of service lines, cafeteria seating capacities, and type of cooking facility.

## 2. Materials and Methods

These study data are from NYC school system, the largest school district in the United States [17]. NYC public school students are very diverse with 41% Hispanic, 26% black, 16% Asian, and 15% white [17]. NYC can be divided into five boroughs (Manhattan, the Bronx, Brooklyn, Queens, and Staten Island), and although it is one school district, the NYC school system consists of 32 community school districts.

### 2.1. Data

This is a secondary data analysis using 2013–2014 administrative data from the NYC Department of Education (DOE) and original survey data we collected from school food managers during the spring of 2014. Five datasets were merged to create one database with observations at the school and building level. The administrative datasets from the NYC DOE include (1) building-level facilities and utilization data from the School Construction Authority (SCA) indicating the SCA defined building capacity and current number of students utilizing the building, (2) building-level cafeteria capacity and (3) meal times data from the Office of School Food, and (4) school-level student demographic data. Finally, school-level descriptors on open campus policies, the number of service lines, type of service lines, and kitchen type were obtained from a survey of school food managers who are the school-level managers of school nutrition programs. Forty-six percent of NYC schools responded to the survey (*n* = 821), and there were no significant differences in breakfast participation, lunch participation nor free/reduced price lunch eligibility between respondents and non-respondents to the survey.

Some NYC schools are multisite, which means that one school is housed across more than one building. Multisite differs from co-location, which occurs when two or more schools are housed in one building. To address the multisite issue, this analysis focuses on the main school building, which is the building with the highest student enrollment, to ensure that each school is accounted for only once in the analysis. Alternative schools were excluded from the study sample. All other 2013–2014 NYC public schools were included in the analysis (*n* = 1804).

### 2.2. Outcome Variables

This analysis includes four outcome variables. The two outcome variables for research question one are breakfast and lunch average daily participation (ADP), calculated as the total number of meals served in 2013–2014 divided by the product of the number of meal service days and average student attendance in 2013–2014. The outcome variable for research question two is a binary indicator for untraditional lunchtimes (lunch before 11:00 a.m. or after 1:30 p.m.). The outcome variable for research question 3 is a binary indicator for an open campus policy. When possible, each model was run separately for elementary, middle, and high schools due to the differences in school meal participation across school levels [3,11,18]. There was not enough variation in open campus status to accommodate separate analysis by elementary, middle, and high schools for research question three.

There is no universal definition for untraditional meal times. Story, Kaphingst, and French (2006) posited 10:00 a.m. and 1:30 p.m. as examples of untraditional lunchtimes [4]. In a 2017 study in New Orleans, Louisiana, 10:45 a.m. was posited as an untraditional lunchtime [19]. A 2014 qualitative report on the ramifications of NYC school co-location suggested 10:30 a.m. as an untraditional lunchtime [14]. Veltman et al. used NYC Department of Education data for an investigation of lunchtimes in NYC public schools for a popular press website, and they used periods occurring before 11:00 a.m. as the cut-off for untraditional lunch periods [20]. In this study, lunch periods that begin before 11:00 a.m. and at 1:30 p.m. or later are defined as untraditional.

### 2.3. Explanatory Variables

There are 11 explanatory variables in this study: a binary variable for school co-location status, a binary variable for overcrowding at the building level (defined as school buildings with >100% of building utilization), six binary variables for service line type (portable service line, double service line, t-line, wide roll-up gate, narrow roll-up gate, and/or straight roll up gate (Figure 1), one continuous variable for the total number of enrolled students per service line, one continuous variable of the ratio of total enrolled students in the building to the cafeteria capacity, and one binary variable for kitchen type (full cooking kitchen or satellite kitchen). Satellite kitchens do not have the capacity to cook foods; instead, they receive delivered food that was prepared in an off-site location and serve this food to students. Service line types (Figure 1) are not necessarily mutually exclusive, as some schools may have multiple line types. The enrolled students to cafeteria capacity ratio was calculated by dividing the sum of the total enrollment of all the schools utilizing the cafeteria by the cafeteria capacity. Full cooking kitchens and satellite kitchens are mutually exclusive.

A correlation matrix was used to better understand the relationships between the explanatory variables. There was only one variable pair that had a moderately strong correlation: the number of students per service lines and student enrollment to cafeteria capacity ratio (r = 0.54). All other correlation coefficients were less than 0.31.

### 2.4. Statistical Analyses

Multivariate beta and logistic regressions were conducted to examine the relationship between each outcome variable and the explanatory variables. To address research question 1, two multivariate beta regressions were used, one for breakfast ADP and one for lunch ADP. For research questions 2 and 3, multivariate logistic regressions were used. In all four regression models, the following covariates were included to isolate the relationship between the outcome and explanatory variables: community school district (of which there are 32 in NYC), percentage of students eligible for free or reduced lunch, percentage of student composition represented by white, black, Asian, and other race students, percentage of Hispanic students, percentage of students with limited English proficiency, percentage of female students, a binary variable for charter schools, and a binary variable for special education schools. A binary variable for Universal Free Meals participation was also included in all four models. At schools designated with Universal Free Meals, all students receive free lunch regardless of family income. Partial and school-wide participation in the Breakfast in the Classroom (BIC) program was controlled for in the regression models for Breakfast ADP. BIC is a program that serves breakfast directly to students in the classroom at the start of the school day, instead of serving breakfast in the cafeteria before the school day begins [21]. The above covariates were selected based on their associations with school nutrition program participation [5,6,18,21,22,23,24].

Stata (version 12.0, College Station, TX, USA) was used for all data cleaning, management, and statistical analysis. This study was approved by the New York University Committee on Activities Involving Human Subjects under the exempt category.

## 3. Results

In 2013–2014, 854 NYC public schools (48.7%) had untraditional lunchtimes. Untraditional lunch times were most common in Queens and least common in Manhattan. Mean cafeteria capacities and number of service lines are also reported in Table 1. On average, the total student enrollment was 2.56 times the cafeteria capacity (range 0.164 to 10.20), and there were 720.33 enrolled students per service line (range 74.0 to 3877.0). Double service lines were the most common service line type and narrow rolling gates were the least common. Of the 741 schools that provided data on open campus policies in the food service manager survey, 66 schools (8.91%) indicated that they have open campuses (Table 1). Fewer than 2% (*n* = 7) of elementary schools reported having an open campus, compared to 8.6% (*n* = 17) of middle and 28.1% (*n* = 39) of high schools. Open campus was most common among Manhattan schools and least frequent among Staten Island schools.

Table 2 shows the multivariate beta regression results for Breakfast and Lunch ADP by grade span. First, co-location status of schools proved to be a significant predictor for school meal participation, as the breakfast ADP in co-located high schools was significantly lower compared to the breakfast in non-co-located high schools (β = −0.056, *p* = 0.003). Second, overcrowding status was also found to be a significant predictor of ADP. For instance, breakfast ADP was found to be lower in elementary (β = −0.046, *p* = 0.03) schools but higher in middle (β = 0.051, *p* = 0.04) and high (β = 0.042, *p* = 0.04) schools that are overcrowded than similar schools that are not overcrowded. Likewise, lunch ADP in crowded elementary schools (β = −0.031, *p* = 0.01) was lower compared to their counterparts. Third, the type of serving line was a significant predictor for ADP. For example, lunch ADP was higher in high schools that have a double service line compared to ones that did not (β = 0.055, *p* = 0.03). ADP for both breakfast (β = 0.072, *p* = 0.04) and lunch (β = 0.058, *p* = 0.03) was higher in middle schools with a T-line service line compared to middle schools without a T-line service line. Lunch ADP was lower in high schools that have a narrow rolling gate compared to high schools that did not have this type of service line (β = −0.070, *p* = 0.02). Additionally, breakfast ADP was higher in elementary (β = 0.052, *p* = 0.05) and middle (β = 0.072, *p* = 0.04) schools with a straight rolling gate than in similar schools without a straight rolling gate. Fourth, enrollment-to-capacity ratios are significantly associated with ADP. Elementary schools with higher enrollment to cafeteria capacity ratios have lower participation in their school breakfast (β = −0.025, *p* = 0.02) and lunch (β = −0.015, *p* = 0.001) programs. However, high schools with higher enrollment to cafeteria capacity ratios have higher lunch participation (β = 0.014, *p* = 0.02). Fifth, the number of students per service line was also significantly associated with ADP. Although marginal, lunch participation was lower in middle (β = −0.0001, *p* = 0.001) and high (β = −0.0001, *p* = 0.001) schools that have a higher number of students per service line compared to similar schools that do not.

Table 3 reports the logistic regression results for untraditional meal times. First, elementary schools in overcrowded buildings have significantly higher odds of having untraditional lunch times compared to elementary schools that are not located in overcrowded buildings (OR = 2.47, CI: 1.05, 5.83), but the same association did not exist in middle (OR = 0.98, CI: 0.29, 3.35) and high schools (OR = 2.18, CI: 0.63, 7.48). Second, middle schools with higher enrollment-to-capacity ratios were more likely to have untraditional meal times (OR = 1.66, CI: 1.03, 2.68), though this relationship was not significant in elementary (OR = 1.06, CI: 0.72, 1.55) or high schools (OR = 1.08, CI: 0.81, 1.44). Third, service line type predicted untraditional lunchtimes. For example, high schools with wide rolling gate service lines were significantly more likely to have untraditional lunch times compared to high schools without this service line type (OR = 4.87, CI: 1.17–20.22). Finally, it is worth noting that co-located schools were no more likely than non-co-located schools to have an untraditional lunchtime, after controlling for other school and cafeteria characteristics in the model.

Table 4 reports the logistic regression results for open campus status. Most notably, co-located schools had significantly higher odds (OR = 2.84, CI: 1.11, 7.26) of having an open campus compared to schools that were not co-located. Schools with a portable service line also had higher odds (OR = 4.33, CI: 1.34, 13.99) of having an open campus compared to schools who did not have this service line.

## 4. Discussion

This study identified several characteristics of schools and cafeterias that are systematically related to meal program participation, untraditional lunchtimes and open campuses. In NYC, schools are required to adhere to strict nutrition standards, but other aspects of school nutrition programs, like open campus policies and untraditional lunch times, are not guided by policy and are under each principal’s discretion. Untraditional lunch times are fairly common, with nearly half of NYC public schools having lunch periods that either begin before 11 a.m. or after 1:30 p.m. Only a fraction of NYC public schools surveyed reported having an open campus, but the practice was more common among high schools. School co-location was significantly associated with breakfast ADP and open campus policies, but not with untraditional lunchtimes. School overcrowding status and the number of students per cafeteria capacity were also associated with breakfast and lunch ADP and untraditional lunchtimes, but not with open-campus policies. Additionally, students per service line was associated with only lunch ADP. Most service line types significantly predicted breakfast ADP and lunch ADP. For untraditional lunchtimes and open campus status, some service line types proved to be not only significant but also more influential than other significant predictors, such as students per cafeteria capacity, students per service line, overcrowding and school co-location. Kitchen type was not significant for breakfast ADP, lunch ADP, untraditional lunch times and open campus status. The relationship between infrastructure characteristics and school meal outcomes varied by grade span, suggesting key differences in infrastructure needs across grade levels.

In 2020, Koch et al. evaluated the cafeteria re-design of 3 NYC high school buildings, two of which were co-located for a total of 7 schools re-designed [25]. The cafeteria re-design consisted of replacing service lines with open, choice-based service zones that served a variety of pre-plated food options, renovating the dining area to provide a variety of table and seating styles (booths, coffee tables, and sofas), new wall décor to improve aesthetics, and signage that promoted nutrition and the menu options. Students’ attitudes towards the service line, dining/seating, and aesthetics improved after the renovation, and average daily lunch participation increased by 14 percentage points 3-months post re-design, which was sustained 1-year after the intervention. Koch et al. did not disclose whether the re-design influenced the school’s open campus policy [25]. In the present study, a double service line and a narrow rolling gate both predicted lunch ADP in high schools, a T-line service line predicted both breakfast and lunch ADP in middle schools, and a straight rolling gate predicted breakfast ADP in elementary and middle schools. Koch et al. [25] and others [5] have demonstrated the link between lunch line length and meal participation among high school students, and narrow rolling gates may be less efficient or may appear less efficient in terms of time spent in line than other line types. Since open campuses were most common among high schools, cafeteria appearance and perceptions of efficiency may be relevant for the choice of school meal participation versus off-campus eating.

Previous research has not examined the influence of service line type and open campus status or untraditional meal times. In this study, portable service lines were associated with increased odds of a school having an open campus policy, suggesting that school principals may respond to a less efficient portable service line by allowing students to eat off campus. This finding is consistent with a 2009 survey of school principals and school district leaders who reported that their current cafeteria facilities were inadequate to serve their entire student body without the option to eat off campus [12]. An alternative explanation may be that administrators are less willing to invest in non-portable service infrastructure in open campus schools.

The number of students per cafeteria seating capacities were positively related to lunch participation in high schools. However, they were inversely related to both breakfast and lunch participation in elementary schools, but the magnitude of this relationship was slightly stronger at breakfast. Cafeteria space may be more important during breakfast periods since morning line-up—when students wait in the cafeteria for their teachers to accompany them to their classrooms—typically occurs in the cafeteria during the breakfast period in NYC schools. Morning line-up is most common in elementary schools, and school breakfast participation is also highest among elementary schools [11]. Morning line up may constrain the available cafeteria space during the breakfast period but would not impact the demand for cafeteria space during lunch.

Overcrowded buildings were associated with untraditional lunchtimes, and this relationship only existed among elementary schools. Elementary students may have less choice during their lunch periods, forcing nearly all of the students to stay in the cafeteria during lunch. In contrast, middle and high school students may be able to attend study hall or club meetings in other parts of the school building during meals, making constrained cafeteria space less of an issue for older grades. This suggests that other factors besides space constraints are more influential drivers of untraditional meal times for older grades.

Although qualitative studies have implicated co-location as a driving factor for untraditional lunch times [14,26], the current study did not support this premise. However, co-location predicted breakfast participation in high schools. Co-location was positively associated with schools having an open campus, while overcrowding and enrollment to cafeteria capacity ratios were not. These findings suggest that an open campus may be viewed as a solution for issues with shared cafeteria space during lunch periods besides constrained cafeterias and overcrowded buildings. For example, staffing complications may occur with shared cafeteria space; sharing outdoor recess space may also be difficult.

This study has some important limitations. First, the cross-sectional design does not allow for causal inference, so the findings should be taken as exploratory and descriptive. Second, we lacked data on open campus policies and cafeteria features for schools that did not respond to our survey. (Our response rate was 45.6%, although responding schools did not appear to be notably different from non-responding schools). Since school food surveys are often done at the district level, previous research does not allow for strong response rate comparisons. School Food Authorities were the target population for the Kitchen Infrastructure and Training in Schools (KITS) survey, which had a response rate of 54.3% [2]. Third, a lack of variation in the open campus variable resulted in some observations being dropped from the logistic regression models, resulting in 19.6% (*n* = 353) of schools represented in the regression model for open campus. Still, at 353 this subset of NYC schools is larger than most U.S. school districts. Fourth, our survey asked about service lines present in the school; it is possible that some lines are present but not utilized.

Additionally, there may be factors that influence open campus or untraditional meal times that were not accounted for in these regression models. For instance, staffing challenges may drive open campus policies. Fewer students on campus during lunch periods would require less staff supervision. Additionally, some schools may not be located near food establishments, making open campus policies less feasible for these schools. This may explain why Manhattan schools most commonly reported having open campus policies since this is the most densely populated borough and most likely to have food establishments located in close proximity to schools. Lastly, nearly all schools in the survey sample had full cooking kitchens, limiting the variation required for this variable to be included in some of the regression models.

The cafeteria re-designs evaluated by Koch et al. cost $750,000 per cafeteria [25], underscoring the high fiscal cost of retrofitting aging buildings to fit the needs of modern, often crowded or co-located urban public schools. While this financial investment may not be possible in all school districts, the present study’s results suggest that school cafeteria space constraints should be considered when school authorities are determining school space, renovation, and new construction allocations. Furthermore, innovative strategies may be needed to work around these space constraints. For example, some Northern Colorado schools have instituted meal counts, where students determine what entrée item they will select during their first period class and require students to get in the lunch line according to which entrée they ordered, to make crowded lunch lines more efficient [27]. School meal programs in other countries, like Japan, offer classroom-based lunches that include educational goals to promote healthy minds and bodies [28]. It is likely that changes in school infrastructure would need to be accompanied by complementary educational endeavors, like those of Japan, in order to promote large shifts in children’s eating behaviors.

## 5. Conclusions

School nutrition programs play a key role in promoting child nutrition security, and school infrastructure challenges may limit the impact of federal nutrition policies like the Healthy, Hunger Free Kids Act. This study finds that service line type, number of students per service line, number of students per cafeteria capacity, overcrowding status and co-location status are systematically associated with school meal participation, but these relationships differ for breakfast and lunch in elementary, middle and high schools. Untraditional lunch times were more likely to occur in overcrowded elementary schools and middle schools with high student enrollment to cafeteria capacity ratios. Co-located schools were more likely to have open campuses. Overall, service line type had the strongest association magnitude with the outcome variables, underscoring the importance of infrastructure characteristics on meal participation, untraditional lunchtimes, and open campus policies. This study’s demonstrated link between school infrastructure characteristics and school meal outcomes suggests that school infrastructure may have public health implications; the potential for constrained cafeterias and service lines should be considered in school space allocation and construction policies. Further research should determine other factors that impact decisions of school principals to allow open campus privileges and untraditional lunchtimes and whether school infrastructure characteristics can be leveraged with other nutrition education and promotion initiatives to influence student consumption and waste behaviors.

## Figures and Tables

**Table 1:** Descriptive Statistics of School Infrastructure and Meal Program Characteristics among New York City Public Schools.

Binary Variables	*n*	Share of Schools ^1^						
		Freq.	Pct. (%)	Manhattan	Bronx	Brooklyn	Queens	Staten Island
				Frequency (%) by Borough				
Untraditional lunch times	1754	869	49.54	132 (39.05)	209 (48.27)	249 (44.70)	231 (65.81)	48 (64.00)
Open campus	741	66	8.91	18 (19.15)	18 (11.18)	14 (5.93)	14 (6.54)	2 (5.56)
Co-location	1802	1076	59.71	244 (68.54)	308 (69.53)	357 (62.52)	148 (41.46)	19 (25.33)
Cooking kitchen	818	812	99.27	145 (97.97)	166 (100.00)	288 (100.00)	179 (98.35)	34 (100.00)
Portable service line	818	134	16.38	44 (29.53)	24 (14.29)	42 (14.58)	23 (12.85)	1 (2.94)
Double service line	818	272	33.25	47 (31.54)	69 (41.07)	79 (27.43)	64 (35.75)	13 (38.24)
T-line	818	195	23.84	31 (20.81)	27 (16.07)	92 (31.94)	41 (22.91)	4 (11.76)
Wide rolling gate	818	117	14.30	24 (16.11)	42 (25.00)	29 (10.07)	18 (10.06)	4 (11.76)
Narrow rolling gate	818	77	9.41	4 (2.68)	27 (16.07)	24 (8.33)	20 (11.17)	2 (5.88)
Straight rolling gate	818	133	16.26	20 (13.42)	24 (14.29)	47 (16.32)	32 (17.88)	10 (29.41)
**Continuous Variables**	***n***	**Mean (SD)**	**Range**	**Mean (SD) by Borough**				
Cafeteria capacity	1732	411.58 (221.75)	0–2000	376.58 (201.89)	463.04 (262.13)	415.62 (214.26)	381.95 (185.15)	383.60 (212.27)
Students per cafeteria capacity	1691	2.56 (1.34)	0.16–10.20	2.90 (1.61)	2.63 (1.30)	2.27 (1.25)	2.65 (1.16)	2.27(0.96)
Number of service lines	819	1.50 (0.68)	1–5	1.43 (0.57)	1.71 (0.90)	1.42 (0.54)	1.52 (0.70)	1.38 (0.55)
Students per service line	810	720.33 (429.16)	74–3877	783.90 (369.97)	743.66 (508.51)	707.22 (438.08)	685.24 (376.64)	623.32 (409.51)
Breakfast Average Daily Participation (%)	1756	26.47 (20.38)	0.00–118.18	24.01 (21.84)	30.46 (20.64)	27.53 (21.00)	22.41 (16.84)	25.76 (17.86)
Lunch Average Daily Participation (%)	1756	65.90 (23.71)	0.52–133.14	56.34 (27.49)	71.21 (20.00)	68.26 (24.29)	66.40 (20.19)	58.54 (22.14)

^1^ Share of schools that possess the characteristics listed under binary variables. Untraditional lunch times are defined as periods that begin before 11:00 a.m. or after 1:30 p.m. Cafeteria capacity and number of service lines are unstandardized. SD = Standard deviation.

**Table 2:** Multivariate Beta Regression Results (Average Marginal Effects) for Percent Breakfast and Lunch Average Daily Participation (ADP) by School Level in New York City Public Schools.

Variables	*Elementary*	*Middle*	*High*	*Elementary*	*Middle*	*High*
	Breakfast ADP	Breakfast ADP	Breakfast ADP	Lunch ADP	Lunch ADP	Lunch ADP
Co-Location Status	0.030	−0.031	−0.056 **	0.002	−0.026	−0.033
	(0.017)	(0.019)	(0.019)	(0.010)	(0.019)	(0.029)
Building Overcrowded	−0.046 *	0.051 *	0.042 *	−0.031 **	−0.006	0.015
	(0.021)	(0.025)	(0.020)	(0.011)	(0.020)	(0.022)
Portable Service Line	0.018	0.043	0.013	0.020	−0.007	0.060
	(0.026)	(0.043)	(0.025)	(0.016)	(0.034)	(0.032)
Double Service Line	−0.017	0.002	−0.024	−0.021	−0.0004	0.055 *
	(0.026)	(0.031)	(0.020)	(0.015)	(0.024)	(0.025)
T-line Service Line	0.022	0.072 *	0.020	0.011	0.058 *	0.045
	(0.025)	(0.035)	(0.025)	(0.013)	(0.026)	(0.030)
Wide Rolling Gate	0.018	0.027	−0.002	−0.00005	0.003	−0.006
	(0.025)	(0.028)	(0.022)	(0.014)	(0.019)	(0.026)
Narrow Rolling Gate	0.021	0.034	−0.008	0.013	0.023	−0.070 *
	(0.034)	(0.027)	(0.032)	(0.019)	(0.025)	(0.030)
Straight Rolling Gate	0.052 *	0.072 *	0.014	0.003	0.033	0.021
	(0.026)	(0.035)	(0.036)	(0.015)	(0.027)	(0.032)
Students per Cafeteria Capacity	−0.025 *	−0.004	0.007	−0.015 **	0.006	0.014 *
	(0.011)	(0.009)	(0.006)	(0.005)	(0.008)	(0.006)
Students per Service line	−0.0001	−0.0001	−0.00003	−0.00002	−0.0001 ***	−0.0001 ***
	(0.0001)	(0.00004)	(0.00002)	(0.00002)	(0.00003)	(0.00002)
Cooking Kitchen		−0.119	0.037		0.034	−0.060
		(0.079)	(0.045)		(0.052)	(0.045)
*n*	300	221	212	296	221	212

Robust standard errors in parentheses. Levels of Significance: *** *p* < 0.001, ** *p* < 0.01, * *p* < 0.05. Each regression is adjusted for school-level demographics (race, ethnicity, sex, free/reduced priced lunch eligibility, limited English proficiency), charter school status, special education school status, participation in the Universal Free Meals program, and community school district). Breakfast ADP regressions are also adjusted for school-wide and partial participation in the Breakfast in the Classroom program.

**Table 3:** Logistic Regression Results for Untraditional Lunch Times by School Level in New York City Public Schools.

Variables	*Elementary*	*Middle*	*High*
	Untraditional Lunch Time	Untraditional Lunch Time	Untraditional Lunch Time
Co-Location Status	0.96	0.44	0.32
	[0.46, 2.04]	[0.19, 1.01]	[0.08, 1.27]
Building Overcrowded	2.47 *	0.98	2.18
	[1.05, 5.83]	[0.29, 3.35]	[0.63, 7.48]
Portable Service Line	1.87	0.25	3.68
	[0.64, 5.46]	[0.04, 1.43]	[0.78, 17.39]
Double Service Line	2.93 *	0.63	4.01 *
	[1.03, 8.35]	[0.19, 2.17]	[1.11, 14.47]
T-line Service Line	2.84 *	0.97	0.70
	[1.02, 7.93]	[0.24, 3.96]	[0.17, 2.92]
Wide Rolling Gate	1.25	0.68	4.87 *
	[0.46, 3.39]	[0.20, 2.30]	[1.17, 20.22]
Narrow Rolling Gate	1.24	0.61	1.01
	[0.30, 5.14]	[0.15, 2.58]	[0.20, 5.15]
Straight Rolling Gate	1.23	0.46	3.69
	[0.39, 3.90]	[0.11, 1.89]	[0.64, 21.13]
Students per Cafeteria Capacity	1.06	1.66 *	1.08
	[0.72, 1.55]	[1.03, 2.68]	[0.81, 1.44]
Students per Service line	1.00	1.00	1.00
	[1.00, 1.00]	[1.00, 1.00]	[1.00, 1.00]
Cooking Kitchen	(omitted) ^†^	(omitted) ^†^	0.44
			[0.02, 12.04]
*n*	302	208	204

Odds Ratios/Exponentiated coefficients; 95% confidence intervals in brackets. Levels of Significance: * *p* < 0.05. Each regression is adjusted for school-level demographics (race, ethnicity, sex, free/reduced priced lunch eligibility, limited English proficiency), charter school status, special education school status, participation in the Universal Free Meals program, and community school district). ^†^ Variable omitted due to lack of variation.

**Table 4:** Logistic Regression and 95% Confidence Intervals Results for Open Campus in New York City Public Schools.

Variables	Open Campus
Co-Location Status	2.84 *
	[1.11, 7.26]
Building Overcrowded	1.87
	[0.65, 5.37]
Portable Service Line	4.33 *
	[1.34, 13.99]
Double Service Line	1.23
	[0.43, 3.49]
T-line Service Line	2.27
	[0.71, 7.20]
Wide Rolling Gate	2.40
	[0.75, 7.71]
Narrow Rolling Gate	1.32
	[0.37, 4.73]
Straight Rolling Gate	3.41
	[0.93, 12.50]
Students per Cafeteria Capacity	1.10
	[0.77, 1.57]
Students per Service line	1.00
	[1.00, 1.00]
Cooking Kitchen	(omitted) ^†^
*n*	353

Odds Ratios/Exponentiated coefficients; 95% confidence intervals in brackets. Levels of Significance: * *p* < 0.05. Each regression is adjusted for school-level demographics (race, ethnicity, sex, free/reduced priced lunch eligibility, limited English proficiency), charter school status, special education school status, participation in the Universal Free Meals program, and community school district). ^†^ Variable omitted due to lack of variation.

## Data Availability

Please contact corresponding author.
